# Utility of Orthodontic Braces as Flexible Splint for Stabilizing an Avulsed Tooth: A Case Report

**DOI:** 10.1155/2024/3913304

**Published:** 2024-09-26

**Authors:** J. Leidenz, Sarah C. Soto Perez

**Affiliations:** ^1^ Dentistry Deparment Kings Dental Center, Doha, Qatar; ^2^ Dentistry Deparment Kings Dental Center, Al Khor, Qatar

**Keywords:** orthodontic braces, splint, tooth avulsion

## Abstract

Dental avulsion is a traumatic dental injury with complete displacement of the tooth outside the bony socket. Both primary and permanent teeth are predisposed to such traumatic incidents. The most effective treatment in such cases is tooth replantation. Additionally, a short-term flexible splint is needed to stabilize the avulsed/replanted tooth. This case report describes orthodontic braces as a standardized flexible splint option for the treatment of tooth avulsion. Herein, the case of a 9-year-old female patient who presented to a private dental clinic with an avulsed upper left central incisor, 1 h after the accident has occurred. Once the tooth was replanted with all the necessary precautions, orthodontic braces and wire were utilized to stabilize the tooth. Most dental splints are custom-made, requiring thorough professional knowledge and expertise for their fabrication and placement. The protocol described in this report is aimed at easing tooth stabilization using materials within the scope of a basic dental setup consultation room.

## 1. Introduction

Dental avulsion is a traumatic dental injury (TDI) with complete displacement of the tooth outside the bone socket [1]. The treatment and prognosis of dental avulsion depends on the apex maturity (open or closed), the duration of the time during which the tooth remains outside the bone socket, and the medium in which it is stored and transported [2, 3]. TDIs have a worldwide prevalence of 15.2% in the permanent dentition, and they are detected in 85% of the patients with traumatic injuries to the oral tissue [4].

Professionals' formation and experience on conditions for TDI treatment [5], evidence-based clinical strategies knowledge, like International Association of Dental Traumatology (IADT) guidelines and Dental Trauma Guide, are essential for avulsed tooth/teeth survival [6, 7]. The chances of dentists and emergency physicians lacking the knowledge of treating TDI [8, 9] can lead to the possibility of failure in avulsion management, splint-type selection, and fabrication, resulting in compromised outcomes.

Whenever possible, immediate tooth replantation should be the treatment of choice for avulsion, regardless of apex maturity and the medium in which the tooth has been transported. If more than 60 min has elapsed since the traumatic event, the prognosis is unfavorable. When performing the tooth replantation, it is recommended to manage the soft tissue, alveolar bone, and root surface by gentle cleaning, 4–6 weeks of flexible splinting, and endodontic treatment after 7–10 days [10]. This approach minimizes the main healing problems, pulp necrosis, and external root resorption [11].

A short-term flexible splint is essential to stabilize the avulsed tooth/teeth. Orthodontic braces (OBs) can prove to be a feasible option because of esthetic, respect for gingival tissue, and buccal placement to the affected and supporting dental tissue through acid-bond adhesive technique. Furthermore, OBs allow slight dental movement within the bony socket, placement of the tooth back to its original position without excessive load, ease of oral hygiene maintenance, and convenient access to thermal sensitivity test and rubber dam isolation for root canal treatment (RCT) [12–14]. Limited literature describes OB as a flexible splint for management of dental avulsion [15]; hence, this case report can be beneficial in the evaluation of OB as a flexible splint for tooth stabilization in avulsion treatment.

## 2. Case Report

A 9-year-old female Arab patient was presented to a private dental clinic in Doha, Qatar; in her mother's words, “she fell and lost the upper left central tooth while running at home, approximately 1 h ago.” The avulsed tooth was stored and transported in a napkin and immediately handed over to the dentist after arriving at the healthcare facility.

**Figure 1:**
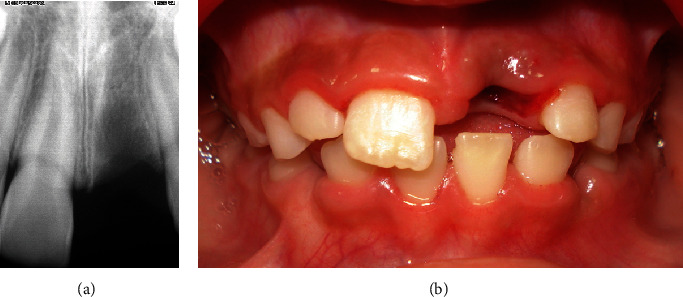
(a) Tooth 21 absence. (b) Bone socket stable blood clot.

Once in a professional's hands, the avulsed tooth was held by the crown with sterile gauze, gently washed with saline, and later immersed in it. The patient's medical history was unremarkable; extra- and intraoral examination revealed a slight bruise in the middle of the upper lip, absent tooth 21, and a stable blood clot in the bone socket. Intraoral periapical radiographs of the affected and surrounding areas showed absence of any alveolar bone or teeth fractures (Figures 1(a) and 1(b)).

**Figure 2:**
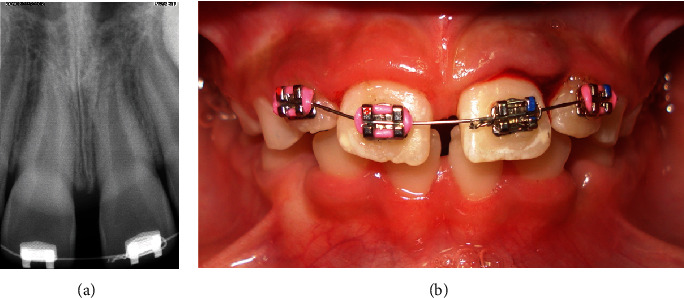
(a) Replanted tooth 21. (b) OBs as flexible split for stabilization.

After reviewing the risks and benefits of the treatment protocol and tooth replantation, the patient's mother agreed and signed a written informed consent. Further, 3% mepivacaine infiltrative anesthesia was applied to the affected area, the bone socket was gently cleaned with saline, and the tooth was replanted without excessive pressure. OBs were buccally bonded to teeth 12, 11, 21, and 22 with a 0.12 stainless steel archwire (Figures 2(a) and 2(b)). The following postoperative medications and instructions were prescribed: Augmentin 250 mg and Cataflam 50 mg, one pill every 8 h for 5 days each, cold and soft diet, and tetanic booster.

**Figure 3:**
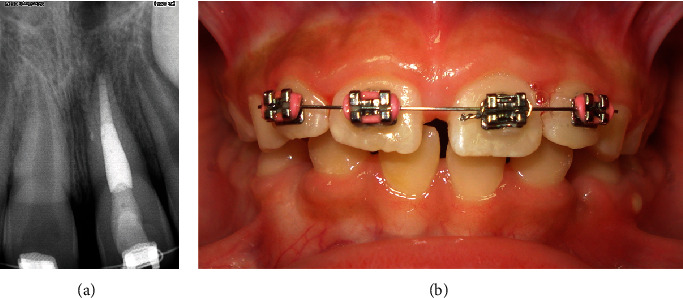
(a) Tooth 21 RCT. (b) At 10 days follow-up.

Ten days later, the patient was asymptomatic and presented normal findings on extra- and intraoral examination. On examination, teeth 12, 11, and 22 were positive to the cold-sensitive thermal test. However, 21 was negative; therefore, pulp necrosis was established as the diagnosis. A single-visit RCT was performed under 4% articaine with 1:100,000 epinephrine as the local anesthesia. The tooth was isolated with a rubber dam sheet, and 3.5% sodium hypochlorite irrigation was performed. A convenient cavity access was made for the root canal system disinfection, the working length was established using the electronic apex locator, and then radiographically corroborated. The apical size preparation was completed with an 80 no. k-file for posterior gray-mineral trioxide aggregate apexification, coronal and middle third warm vertical obturation was performed, and later the tooth was restored with a light-cure composite filling material (Figures 3(a) and 3(b)).

**Figure 4:**
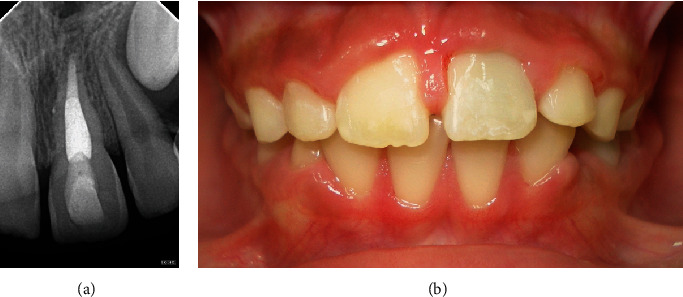
(a) 12-month follow-up, radiographic signs of ankylosis. (b) Clinical normalcy and gum hyperplasia.

The OB flexible splint was removed 6 weeks later once the tooth was stable. The 12-month follow-up showed clinical normalcy and radiographic signs of ankylosis (Figures 4(a) and 4(b)).

## 3. Discussion

Avulsions represent 0.5–16% of all dental traumas [10]. A 19-year traumatic injuries bibliometric study from 1999 to 2018 showed that case reports were the most published literature type, with prevalence of dental avulsion being the highest [16]. Nevertheless, few case reports mentioned [13, 14] and described [12] OB as a flexible splint option.

The 2012 IADT guidelines for permanent teeth avulsion were the present strategies for such traumatic events management [10]. Nevertheless, the aforesaid did not mention prompt tooth attention; hence, minutes passed by, making the prognosis less favorable. The current case report presented prompt attention being given to both, the patient and the avulsed tooth, as described by the 2020 IADT guidelines for avulsion, placing the avulsed tooth in saline, while the patient was examined and prepared for replantation [16, 17].

Once the avulsed tooth is replanted, a flexible tooth splint is needed to provide stability; this requires time and recommended material, such as nylon [17], which might not be readily available at the time of consultation. Furthermore, the patient's stress due to recent trauma and lack of standardized splitting techniques could affect the treatment prognosis and outcome. In contrast, devices such as the Titanium Trauma Splint (TTS) use a standardized manufactured splint to shorten the working time, minimize manufacturing difficulties, and simplify its placement by bonding it to the buccal teeth surfaces with composite restorative material [18, 19].

A cross-sectional local study found that specialists and general practitioners lack the knowledge of TDI management [20], potentially affecting tooth avulsion treatment's ultimate goal of tooth/teeth survival [21]. Moreover, a recent review stated that further research should focus on selecting dental avulsion splint type and timing [22]. The current case report dives into the proposal by using OB as a flexible splint for stabilization in an upper left central incisor avulsion following IADT management and splinting principles [17].

In this case, as the total extraoral dry time of the tooth exceeded 60 min, the prognosis and outcome were expected to be unfavorable. The 12-month follow-up exhibiting ankylosis (replacement resorption) is an anticipated and compatible outcome in the literature, which states that factors most closely related to periodontal ligament healing are root stage development, tooth extra-alveolar dry time, immediate replantation, and wet storage time [23], similar to animal models [24].

In conclusion, within the case limitations, as seen in the 1-year follow-up, OBs are a feasible, standardized, flexible splint treatment option for the stabilization of dental avulsion. The well-known etch-bond technique in dentistry, as well as the materials and instruments available at clinical dental setups for OB placement, positioning, and debonding, eases the splint manufacturing and removal process for professionals.

## Data Availability

The authors have nothing to report.
